# Effect of O/W process parameters on *Crataegus azarolus* L nanocapsule properties

**DOI:** 10.1186/1477-3155-11-16

**Published:** 2013-05-29

**Authors:** Akbar Esmaeili, Soraya Rahnamoun, Fariba Sharifnia

**Affiliations:** 1 Department of Chemical Engineering, North Tehran Branch, Islamic Azad University, PO Box 19585/936, Tehran, Iran; 2 Department of Chemistry, Pharmaceutical Sciences Branch, Islamic Azad University, Tehran, Iran

**Keywords:** Nanocapsule, *Crataegus azarolus*, Emulsion, Triblock copolymer, Medicinal plant

## Abstract

**Background:**

Nanocapsules have many applications in the drug, cosmetic, fragrance, and food industries. In this study, *Crataegus azarolus* L. nanocapsules were prepared by a modified emulsion diffusion technique.

**Methods:**

In this technique a shell was first made from the polyester triblock copolymer poly(ethylene glycol)-poly(butylene adipate)-poly(ethylene glycol) (PEG-PBA-PEG) and then olive oil was set as the core of the nanocapsule by a method known as the polymer deposition solvent evaporation method. Varying amounts of *C. azarolus* extract, polymer, and olive oil were mixed in acetone and then added to water on a shaker. Finally, the acetone was removed by vacuuming.

**Results:**

The size of the prepared nanocapsules were measured with a particle size analysis report (PSAR) and identified by scanning electron microscopy (SEM), Fourier transform infrared spectroscopy (FTIR), and nuclear magnetic resonance (NMR).

**Conclusions:**

Our experiments showed that the size of the nanocapsules depends on the preparation conditions, i.e., the ratio of polymer to oil and concentrations of polymer and plant extract. A ratio of 1:0.25 polymer to oil was shown to be more suitable for the formation of smaller nanocapsules of *C. azarolus.*

## Background

*Crataegus azarolus* L. is a species of hawthorn that thrives in semiarid conditions and areas with no shade, e.g., in woods and hedges on dry hillsides and mountains. The fruit and flowers of this plant have a hypotensive effect as well as acting as a direct and mild heart tonic. They are especially useful in the treatment of heart conditions associated with high blood pressure. In most European countries, especially Germany, hawthorn, which belongs to the Rosaceae family, is used to treat irregular heartbeat. It can also be applied in the treatment of angina and arteriosclerosis (hardening of the vessel wall). The aromatic fruit is rich in malic and tartaric acid and can be employed in cases of anemia and tuberculosis, because of its tannins and astringent properties. It can also be used to treat bloody diarrhea. It is less commonly prepared for nonmedicinal ingestion.

Generally, nanoparticles are defined as solid colloidal particles and include both nanospheres and nanocapsules. They can be prepared by both polymerization methods and synthesis with preformed polymers [1,2]. Fragrance and flavor play an important role in such products as perfume, soap, cream, food, and cigarettes. Encapsulation is an important method used in solving problems related to imparting fragrance and flavor to commercial products. Encapsulation techniques are used in the food and cosmetic industries to control the release of entrapped materials and to protect against surrounding environments. Fragrance encapsulation and its controlled release play a key role in production of fragrance samples incorporated as films or fine powders on magazine pages, aimed at providing consumers with the opportunity to try a fragrance, and thus enhancing its marketability. Encapsulation stabilizes the fragrance, while controlled release prolongs its longevity. Once the flavor or fragrance is encapsulated, controlled release is accomplished by diffusion, pressure change, temperature sensitivity, or other stimuli. However, fragrances and flavors are complex mixtures of comparatively volatile substances, the labile components of which can be changed as a result of heating, oxidation, chemical interactions, or volatilization, thus altering their sensory perception. Microencapsulation technology is an effective method of minimizing these problems. Encapsulation of fragrances or flavors has been attempted using various methods [3-5].

Block copolymer micelles also possess a number of properties that make them suitable as drug carriers. Firstly, hydrophobic drugs can be encapsulated physically by the core of the block and be carried at rates that exceed their intrinsic water solubility rate. Secondly, the hydrophilic blocks can form hydrogen bonds with the surrounding aqueous medium and form a tight shell around the micellar core [6]. The polymeric shell of the drug provides protection against degradation factors such as pH and light and reduces tissue irritation [7,8].

Nanocapsules can be likened to vesicular systems in which a drug is confined in a cavity consisting of an inner liquid core surrounded by a polymeric membrane [3]. The cavity can contain the active substance in liquid or solid form or as a molecular dispersion [9,10]. This reservoir can be lipophilic or hydrophobic according to the preparation method and the raw materials used [11]. Morphology, particle size, and release properties of nanocapsules are the key considerations in designing nanoparticulate delivery systems. Since the release profile of a drug predominantly depends on the nature of the polymer and the morphology of the nanocapsules, fundamental understanding of the relationship among these key characteristics and release mechanisms is essential to yielding useful products [12]. It has been postulated that the morphology of nanocapsules depends on the rate of polymer solidification and solvent removal at the interface [13]. A smooth surface is generated when the polymer precipitates slowly due to slow removal of the organic solvent [14]. Method and rate of solvent removal, dispersed-phase/continuous-phase ratio, polymer concentration, and stabilizer concentration in inner water phase are critical factors in determining the morphology and surface area of nanocapsules [15].

Nanoencapsulation has also been found to be of use in pharmaceutical and nutraceutical industries as it has been used to affect targeted delivery, minimize toxicity, enhance biodistribution, and increase cell uptake of some drugs and nutraceuticals [16].

Tachaprutinun et al. have recently improved the thermal stability of astaxanthin, an important industrial carotenoid pigment used as food coloring, by polymeric nanoencapsulation [17]. Sane and Limtrakul (2009) have successfully employed nanoencapsulation to protect the vitamin supplement retinyl palmitate from photodegradation induced by UV radiation [18]. Nanoencapsulation has been used to mask the naturally pungent odor of capsaicin, thus enhancing its beneficial utilization [19]. Nanoencapsulation of omega-3-fatty acids is a novel method for protecting them from various deleterious environmental conditions [20]. Esmaeili et al. have investigated the formation of nanocapsules containing *Matricaria recutita* L. extract, created using the emulsion-diffusion process [21].

Generally, there are six classical methods for the preparation of nanocapsules: nanoprecipitation, emulsion-diffusion, double emulsification, emulsion-coacervation, polymer-coating, and layer-by-layer [11]. In this work we have employed the emulsion-diffusion method.

## Materials and methods

### Chemicals

Adipoyl chloride, butylene glycol, ethoxyethane, polyethylene glycol 2000, and triethylamine were obtained from Labscan Ltd., Dublin, Ireland. Other materials obtained locally were acetone and pure water.

### Synthesis of triblock copolymer (PEG-PBA-PEG)

**Figure 1:**
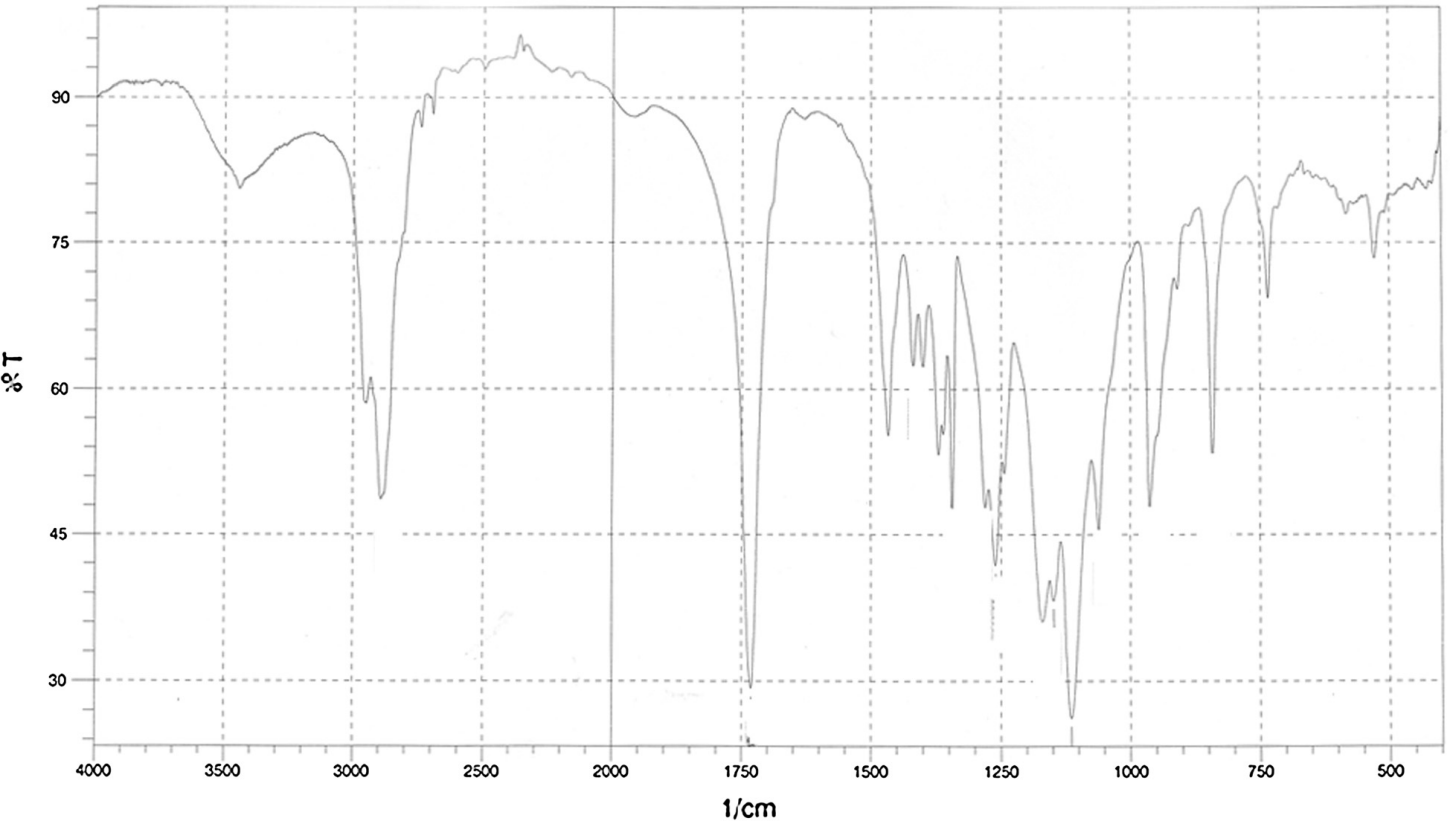
PEG-PBA-PEG FTIR graph.

Synthesis of the triblock copolymer (PEG-PBA-PEG) was started by mixing adipoyl chloride (1.7 mL) and butylene glycol (0.98 mL, 11 mmol) at 85°C in a balloon with a magnetic stirrer and adding three drops of triethylamine. The aggregative polymerization reaction for preparing the polyester continued for about 24 h; after that no HCl was released. In this stage the proportion of alcohol acid chloride polyester to HCl was determined to be 1:1.5. In the stage for the preparation of triblock, an additional amount of PEG (2000) was added to polyester. The reaction was kept at a constant temperature. The end of the reaction was signaled when no further HCl was released in the reaction. The polymer was precipitated using diethyl ether (40 mL) at 15°C then washed three times with distilled water and separated using a centrifuge. Analysis with gel permeation chromatography (GPC) revealed molecular weights of M_W_ = 30.974, M_n_ = 8049, and M_W_/M_n_ = 3.85 (Figure 1).

### Plant materials and extraction and isolation

Aerial parts of *C. azarolus* (300 g) (Voucher No. 2378) containing leaves and flowers were collected from Taleghan, province of Ghazvin, Iran, in May 2012, during the flowering stage. Voucher specimens have been deposited at the Herbarium of the Research Institute of Forests and Rangelands, Tehran, Iran. The aerial parts of *C. azarolus* were extracted with methanol in a soxhlet apparatus over the course of 2 days. The crude residue (55 g) was obtained after filtration and the evaporation of the solvent and washed with n-hexane, dissolved in distilled water, and then successively extracted with AcOEt and BuOH.

### Preparation of core-shell nanoparticles by emulsion-diffusion

Synthesis of PEG-PBA-PEG nanocapsules containing the extract was carried out in the absence of a surfactant*.* First, definite amounts of PEG-PBA-PEG copolymer, *C. azarolus* extract, and olive oil were dissolved in (2 mL) of acetone, yielding an organic solution. This organic solution was stirred drop by drop into 10 mL water at room temperature. A suspension solution was then prepared and exposed to ultrasound and then the acetone removed under vacuum and the water removed by freeze-drying. After this the suspension solution containing nanocapsules was passed through a filter (0.25 μm). This helped in separating the stacked mass without contributing to the extraction of the nanoparticles. The size of the nanoparticles in the suspension solution was measured by PSAR. In this work factors such as concentration of polymer, ratio of polymer to oil, concentration extract, and presence of surfactant were varied in the production of nanocapsules, in order to study the effects of each.

### Synthesis of nanoparticles with different concentrations of polymer

To study the effect of polymer concentration on nanocapsule behavior, four samples containing different concentrations of polymer with a 1:1 weight ratio of oil to polymer were prepared. The amounts of polymer in the different samples were 0.25, 0.5, 1, and 2 mg. The amount of extract remained constant at 1.6 mg, and oil was increased in equal ratio to the polymer. The *C. azarolus* extract, polymer, and oil were dissolved in 2 mL of acetone and added drop by drop to 10 mL of water while being stirred.

### Synthesis of nanoparticles with different ratios of polymer to oil

For studying the effect of the ratio of polymer to oil on nanocapsules, four samples containing different ratios of polymer to oil were prepared by keeping the polymer concentration constant at 0.5 mg and varying the oil concentration to 1.00, 0.50, 0.25, 0.125, and 0.0625 mg. The amount of *C. azarolus* extract used was 1.6 mg. The extract, polymer, and oil were dissolved in 2 mL of acetone and added drop by drop to 10 mL of water at the aqueous phase.

### Synthesis of nanoparticles with different concentrations of *C. azarolus* extract

Four samples with different amounts of extract were prepared for studying the effect of the drug on nanoparticle size. This time the polymer/oil ratio was fixed at 1:0.25, and the concentration of extract was varied. The amount of oil used was 0.125 mg, the amount of polymer was 0.5 mg, and the concentration of the extract used in the varying samples was 0.25, 0.35, 0.45, and 0.65 mg. The extract, polymer, and oil were dissolved in 2 mL of acetone and added drop by drop to 10 mL of water at the aqueous phase.

### Synthesis of PEG-PBA-PEG nanocapsules containing extract in the presence of surfactant

To study the effect of a surfactant on nanoparticle size, fixed amounts of PEG-PBA-PEG copolymer, *C. azarolus* extract, and olive oil were dissolved in 2 mL of acetone to form the organic solution. This was then stirred drop by drop into 10 mL of water containing the surfactant Tween 80 and glycerin 50% at room temperature. The prepared suspension solution was exposed to ultrasound, and after that the acetone was removed under vacuum and the suspension solution containing nanocapsules was diluted three times with deionized water and centrifuged at 6000 rpm for about 1 minute. The prepared nanocapsules were measured by PSAR. The amounts of oil, extract, Tween, and glycerin used were 0.125 mg; 0.25 mg; 0.1, 0.3, and 0.6 mg; and 0.05, 0.15, and 0.3 mg respectively.

## Results and discussion

**Figure 2:**
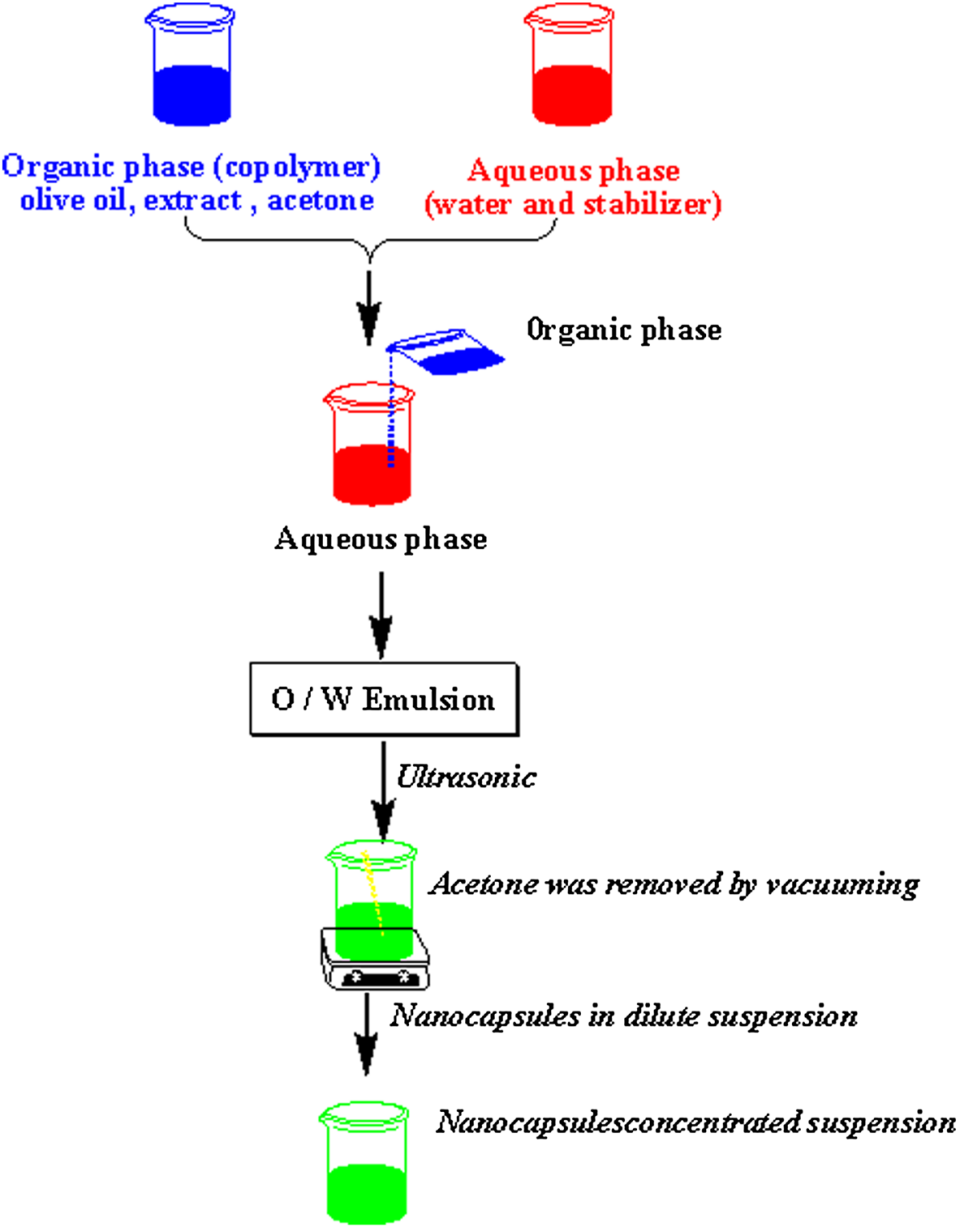
The emulsion-diffusion method.

Nanocapsules consist of a liquid core surrounded by a polymeric membrane; they have a core-shell type internal structure [22]. Nanocapsules can be used as smart drugs that have specific chemical receptors and only bind to specific cells. The advantages of nanoencapsulation technologies for pharmaceutical applications include higher dose loading with smaller dose volumes, more rapid absorption of active drug substances, increased bioavailability of the drug, higher safety and efficacy, and improved patient compliance. According to Quintanar et al. (1998, 2005), nanocapsules prepared with the emulsion-diffusion method can be utilized for both lipophilic and hydrophilic active substances [11]. The emulsion-diffusion method is a double-stage process consisting of (1) emulsion production and (2) solvent diffusion (Figure 2). This method has been employed in other research [23]. As a basis of comparison, we used the same method and studied similar variations in parameters.

**Figure 3:**
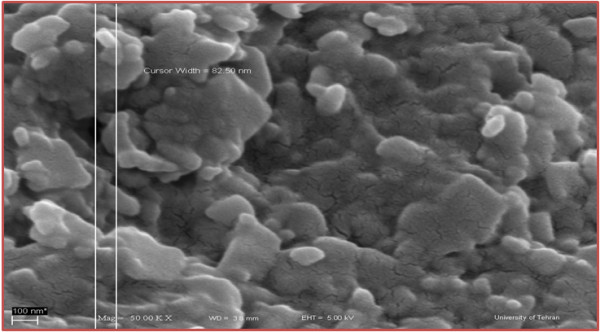
**SEM of nanocapsules containing *C. azarolus *extract.**

In the first stage, an oil-in-water emulsion is fabricated by dissolving the polymer and the oil in an organic solvent. The elimination of the organic solvent contained in the oil phase leads to the separation of the polymer and the oil and a reduction in the particle size. Following this organic phase the process enters the aqueous phase and consequently leads to polymer precipitation around the oil particles, producing nanocapsules. The organic solvents have to dissolve in oil, polymer, and extract, and be partially soluble in water. Acetone is a useful solvent for this purpose. The relationship between the oil viscosity and the droplet size has been thoroughly investigated by researchers for a variety of vegetable oils with differing viscosity and interfacial tension towards the water phase, and it has been shown that olive oil has the highest viscosity and interface. In this research olive oil has been used due to its biocompatibility, similarity with nutraceutical oils, and ability to dissolve hydrophobic compounds [21]. Each emulsion droplet produces several nanocapsules, and these are formed by the combination of polymer precipitation and the interfacial phenomena during solvent diffusion. Nanocapsules have a core-shell structure in which the shell is polymeric and is surrounded by the hydrophilic part of the PEG. An SEM picture of nanocapsule is shown in Figure 3.

### Synthesis of nanoparticles with different concentrations of polymer

**Figure 4:**
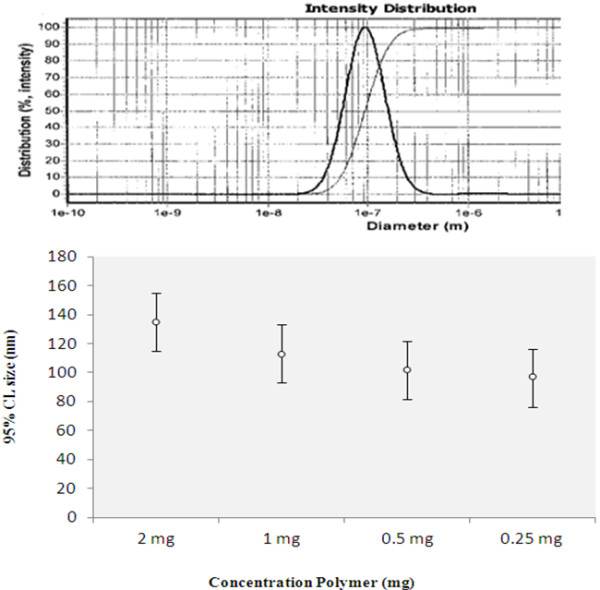
PSAR report graph for 0.5 mg polymer (95.5 nm).

The experimental results show that with increased concentrations of polymer in the organic phase, particle size increases, and with decreased concentration, the size of the nanocapsules decreases. This is because with increased polymer concentration, the polymer-polymer interaction decreases; this causes the separation of the polymer chains during the emulsification process to be more problematic, and thereupon the particle size increases. With increased polymer concentration in the organic phase, the viscosity of the phase rises and as a result the solvent diffusion in the aqueous phase diminishes. This causes the nanoemulsion drops to become larger and increases the particle size; in our study, the samples with 0.5, 1, 2, and 4 mg of polymer yielded particle sizes of 95.2 nm, 113 nm, 134 nm, and 148 nm respectively. The size of the nanocapsules was measured by PSAR. Results are shown in Figure 4 and Table 1.

**Table 1:** Result of particle size analysis report for different concentrations of polymer

**Sample**	***C. Azarolus *extract (mg)**	**Polymer/oil ratio**	**Polymer (PEG-PBA-PEG) (mg)**	**Average size of particle (nm)**
1	1.6	1:1	o.5	95.5
2	1.6	1:1	1	113
3	1.6	1:1	2	134
4	1.6	1:1	4	148

### Synthesis of nanocapsules with different polymer/oil ratios

**Figure 5:**
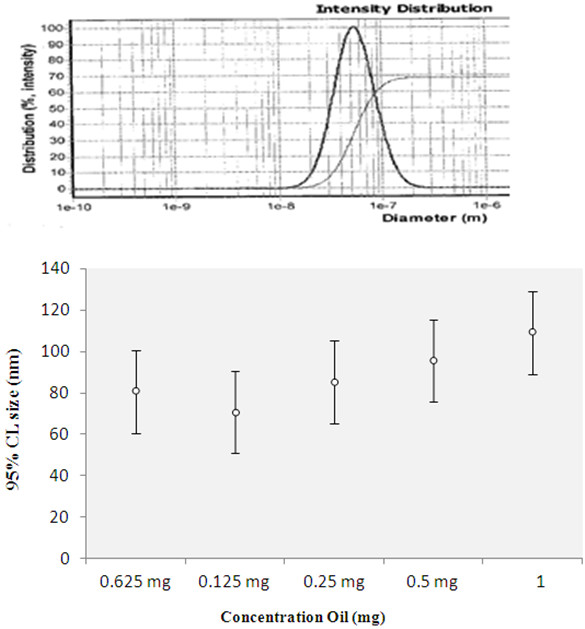
PSAR report graph for 1:0.25 oil/polymer (70.7 nm).

When the amount of oil is increased, the particle size increases. By increasing the amount of oil in the organic phase the viscosity increases and as a result the size of the nanocapsules will increase. Increasing the amount of oil increases the efficiency of the extract and as a result the size and number of nanocapsules increases. When the ratio of oil to polymer reaches 2:1 this factor will decrease. In our study, for polymer/oil ratios of 1:2, 1:1, 1:0.5, and 1:0.25 particle sizes of 108 nm, 95 nm, 85.3 nm, and 70.7 were obtained. The size of the nanocapsules was measured by PSAR. Results are shown in Figure 5 and Table 2.

**Table 2:** Result of particle size analysis report for different ratios of polymer to oil

**Sample**	***C. Azarolus *extract (mg)**	**Polymer/oil ratio**	**Polymer (PEG-PBA-PEG) (mg)**	**Average size of particle (nm)**
5	1.6	1:2	0.5	108
6	1.6	1:1	0.5	95
7	1.6	1:0.5	0.5	85.3
8	1.6	1:0.25	0.5	70.7

### Synthesis of nanoparticles with varying amounts of extract

**Figure 6:**
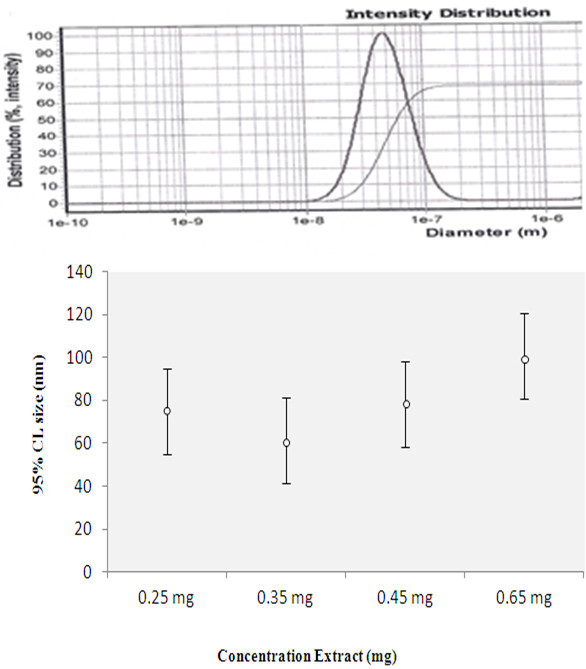
PSAR report graph for 0.35 mg extract in food (61.4 nm).

**Figure 7:**
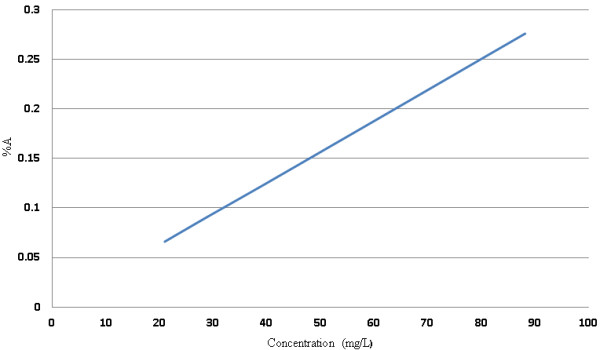
**Molecular absorption curve in λ**_**max **_**670 cm.**

Our results showed that with an increase in the amount of extract, the size of the particle will decrease. This is due to the structure of the polymer. Polymers have a semicrystalline structure that can be very irregular and can cause the formation of crystals in the nanocapsule structure. The interaction between the extract and polymer causes an aggregation of polymer in the nanocapsule structure. The degree to which this occurs depends on the concentration of the extract; when the amount of extract in the nanocapsules increases, the extract acts as a clog factor, causing an increase in the particle size and decreasing the stability [23]. Our results showed particle sizes of 74.2, 61.4, 78.3, and 99.9 nm for 0.25, 0.35, 0.45, and 0.65 mg of extract in the samples. The size of the nanocapsules was measured by PSAR (Figure 6 and Table 3). The molecular absorption curve in λ_max_ = 670 is displayed in Figure 7, which shows that with an increase in the amount of extract, the absorbance increased.

**Table 3:** Result of particle size analysis report for varying amounts of extract

**Sample**	***C. Azarolus *extract (mg)**	**Polymer/oil ratio**	**Polymer (PEG-PBA-PEG) (mg)**	**Average size of particle (nm)**
9	0.25	1:0.25	0.5	74.2
10	0.35	1:0.25	0.5	61.4
11	0.45	1:0.25	0.5	78.3
12	0.65	1:0.25	0.5	99.9

### Synthesis of PEG-PBA-PEG nanocapsules containing extract in the presence of surfactant

**Figure 8:**
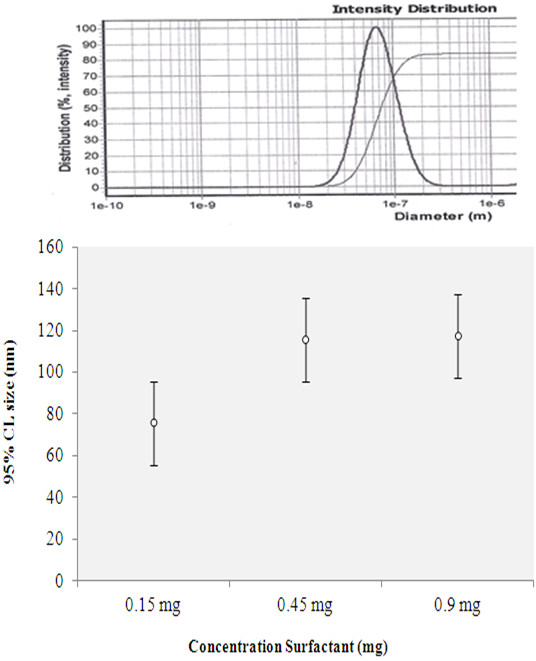
PSAR report graph for 0.1 mg Tween and 0.5 glycerin (75nm).

Surfactants play a key role in the separation of the two phases (oil and water) to form the emulsion. The surfactants stabilize the dispersed phase droplets formed during emulsification; inhibit coalescence of droplets; and determine the particle size, morphological properties, surface composition, and release properties of the nanocapsules [24]. The results showed respective particle sizes 75, 115, 117, and 134 nm for 0.5, 0.1, 0.3 and 0.5, 1.5, and 3 mg of Tween and glycerin. The results are shown in Figure 8 and Table 4.

**Table 4:** Result of particle size analysis report in presence of varying amounts of surfactant

**Sample**	***C. Azarolus *extract(mg)**	**Polymer/oil ratio**	**Polymer (mg)**	**Tween**	**Glycerin**	**Average size of particle (nm)**
9	0.25	1:0.25	0.5	0.1	0.5	75
10	0.25	1:0.25	0.5	0.3	1.5	115
11	0.25	1:0.25	0.5	0.6	3	117

## Conclusions

Nanocapsules loaded with medicinal plants have many applications in drug manufacturing, as well as other industrial and commercial uses. In this research the development of a useful emulsion-diffusion method for preparing nanocapsules loaded with *C. azarolus* was investigated. In our study, we show that nanocapsule size is related to parameters such as polymer concentration, oil-to-polymer ratio, extract concentration, and amount of surfactant. The nanocapsules were identified by PSAR, SEM, FT-IR, and NMR. Because of efficiency of delivery, these nanocapsules would allow for as much as a 10,000-fold decrease in needed drug dosages. With a reduced dosage of the drug, side effects are kept at a minimum level.

## Competing interests

The authors declare that they have no competing interests.

## Authors’ contributions

All authors contributed equally, read and approved the final manuscript.
